# Poly(ethylene glycol)-Based Surfactant Reduces the Conformational Change of Adsorbed Proteins on Nanoparticles

**DOI:** 10.1021/acs.biomac.2c00744

**Published:** 2022-09-09

**Authors:** María Martínez-Negro, Daniela Russo, Sylvain Prévost, José Teixeira, Svenja Morsbach, Katharina Landfester

**Affiliations:** † Max Planck Institute for Polymer Research, Ackermannweg 10, 55128 Mainz, Germany; ‡ Consiglio Nazionale delle Ricerche (Instituto Officina dei Materiali) and INSIDE@ILL c/o Institut Laue-Langevin, 38042 Grenoble, France; § Institut Laue-Langevin, 38042 Grenoble, France; ∥ Université Paris-Saclay, Laboratoire Léon-Brillouin, UMR12 CEA-CNRS, CEA-Saclay, F-91191 Gif-sur-Yvette CEDEX, France

## Abstract

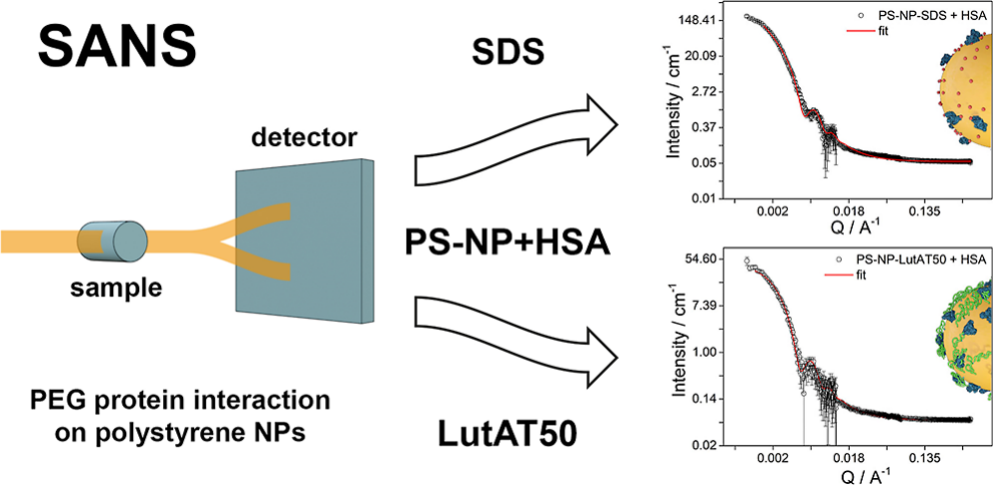

When in contact with a biological medium, the surfaces of nanoparticles are usually covered by proteins. In this regard, it was found that poly(ethylene glycol) (PEG) promotes the “stealth effect”. This implies a reduction of unspecific protein adsorption and cellular uptake. Although information about the PEG–protein interaction was reported, more accurate and sophisticated structure and dynamics analyses are needed to understand the interaction processes in detail. This work studies the PEG–protein interaction using model nanoparticles stabilized either by the PEG-based surfactant Lutensol AT50 or sodium dodecyl sulfate. The interaction with human serum albumin was studied using neutron scattering techniques. The parameters obtained by small-angle neutron scattering yielded information about the adsorbed protein layer thickness. Protein structure changes were detected via differential scanning fluorimetry and elastic neutron scattering. This combination gives a better insight into the PEG–protein interaction, contributing to the design of nanomaterials for medical applications.

## Introduction

Nanocarriers for biomedical applications are usually designed to achieve the desired transport effect avoiding their rapid clearance from the bloodstream and the induction of an immune response. However, after introduction into the body, proteins and other biomolecules present in the blood are adsorbed onto the surface of colloidal nanomaterials.^1,2^ These adsorbed biomolecules constitute the so-called protein or biomolecule corona and influence the biological behavior, including biodistribution, immune response, and elimination from the body.^3^ Hydrophilic polymers such as poly(ethylene glycol) (PEG) are commonly used to promote the so-called “stealth effect”. Initially, it was observed that protein adsorption decreases if nanomaterials are functionalized with PEG chains.^4−6^ This effect leads to longer circulation times in the bloodstream and reduction of unspecific cell uptake. Further studies suggested that the presence of PEG not only reduces the overall unspecific protein adsorption in blood plasma but can also promote the enrichment of particular proteins.^6,7^ These specific proteins would play a critical role in triggering the highly desired stealth effect. Although functionalization with PEG is currently the most commonly used strategy, new polyphosphoester (PPE) polymers such as poly(ethyl ethylene phosphate) are emerging as a biodegradable alternative.^8,9^ A few studies demonstrated that PPE conjugates exhibit similar protein adsorption patterns to those with PEG.^10^ Other examples are cylindrical brush polymers such as polysarcosines, which are suitable carriers that extend in vivo circulation times and show high biocompatibility.^11^ For example, azide groups on the poly oxazoline side chains effectively target dendritic cells and exhibit low immunogenicity in different animal models.^12^ Even though PEG and its developed alternatives are now intensively investigated, it is still unclear which are the molecular scale processes leading to different interactions with proteins. Thus, we intend to understand the protein interactions of PEG on nanocarriers compared to non-PEGylated surfaces. One of the biggest challenges in this regard is the difficulty of finding suitable characterization techniques that can provide the necessary resolution. For example, circular dichroism determines the conformational changes of the secondary and tertiary structure of proteins, but transparent solutions are required.^13^ NMR spectroscopy can provide detailed structural information about macromolecules at an atomic resolution but becomes extremely challenging and resource-demanding for proteins.^14^ Other techniques such as cryogenic transmission electron microscopy offer a powerful view of the protein structure. However, all the techniques mentioned above do not provide structural information at a sufficiently high resolution and are not interface-specific. In contrast, small-angle scattering is a powerful tool to study the structure and interaction of colloids to overcome these disadvantages. For example, the interaction between silica nanoparticles (NPs) and bovine serum albumin indicates that they behave as individual entities due to the electrostatic repulsive interaction.^15^ Likewise, small-angle X-ray scattering and small-angle neutron scattering (SANS) were used together with some theoretical models to evaluate the interaction between gold NPs and human serum albumin (HSA).^16^ On the other side, the interaction between surfactants with proteins and NPs separately have been also reported, that is, fibrillation of α-synuclein protein by sodium dodecyl sulfate (SDS)^17^ or the interaction of anionic silica NPs with ionic and nonionic surfactants.^18^ More complex systems constituted by three components, NPs, proteins, and surfactants, are the next step in understanding the impact of surfactants on protein adsorption.

This work focuses on small-angle scattering because it offers a reliable nanometer-resolution structural characterization of proteins and their interactions as it is able to quantitatively discriminate between adsorbed and dispersed proteins in bulk. SANS and elastic neutron scattering (ENS) can be applied to determine structural parameters and dynamics of protein–NP interactions. Both neutron scattering techniques allow the determination of the low-resolution structure of individual colloidal components such as the nanomaterial functionalization by H/D isotopic substitution. Furthermore, correlation with other techniques, such as nano differential scanning fluorimetry (nanoDSF) gives information about the conformational stability of the proteins. We applied a combination of these techniques to obtain insights into the impact of PEG on model polystyrene (PS) NPs on the protein adsorption. Lutensol AT50 (LutAT50) was selected as a PEG-based surfactant for surface coating and compared with the negatively charged SDS. As a protein, HSA was determined to be the most prevalent protein in human plasma. Thereby, we were able to identify small changes in the thickness of the adsorbed protein layer, which correlate well with the information obtained about the protein structure.

## Materials and Methods

### Samples

HSA was purchased from Sigma-Aldrich (St. Louis, MO; product no. A3782) and used without further purification. All protein solutions were freshly prepared with D_2_O from Sigma-Aldrich (≥99.9 atom % D). Styrene-*d*_8_ (≥98 atom % D) and hexadecane (>99%) were also purchased from Sigma-Aldrich. Styrene was freshly purified before the synthesis via distillation to remove the stabilizer 4-tertbutylcatechol. 2,2′-Azobis(2-methylbutyronitrile) (V59) was purchased from Wako Chemicals (Neuss, Germany). The nonionic surfactant Lutensol AT50 (PEG-hexadecyl ether) was purchased from BASF AG (Ludwigshafen, Germany). The anionic surfactant SDS was purchased from Fluka (Sigma-Aldrich).

### NP Preparation

PS NPs with either SDS or Lutensol AT50 as the surfactant were synthesized using the miniemulsion polymerization method with styrene-*d*_8_ as the monomer according to a previously published procedure.^19^ In brief, the aqueous phase containing Lutensol AT50 or SDS was added to a mixture of styrene-*d*_8_, initiator V59 (Wako Chemicals), and the hydrophobe (hexadecane). After 1 hour of pre-emulsification, the mixture was sonicated using a Branson Sonifier (1/2 in. tip, 6.5 mm diameter) for 2 min at 450 W and 90% amplitude in an ice-cold bath. The polymerization was carried out at 72 °C at 1000 rpm. The resulting NPs were washed five times via centrifugation and resuspension in D_2_O. The NPs were filtered through Millex-SV 5 μm filters (Merck Millipore, Billerica, USA) before use to remove aggregates or potential impurities such as dust. Both NPs were synthetized with a final solid content of 1 wt %.

### Particle Characterization

The hydrodynamic size of the NPs was measured by dynamic light scattering (DLS) using a Zetasizer Nano (Malvern Instruments). The final sample concentration was 5 mg mL^–1^, and all the measurements were run at 25 °C.

### Small-Angle Neutron Scattering

SANS data were collected from D_2_O dispersions of the pure NPs, pure surfactants, native HSA in D_2_O, and mixtures of HSA/NPs. The final concentration for native HSA and for HSA in all the mixtures was 5 mg mL^–1^. The NP–protein mixtures were centrifuged at 20,000*g* for 30 min and subsequently redispersed in D_2_O before the measurements to eliminate all free protein left in solution. SANS data were collected on the D11 SANS instrument (ILL, France). All measurements were performed using an incident neutron wavelength λ = 5 Å and at two different sample–detector distances and acquisition times (2 m for 10 min and 14 m for 30 min). These conditions yield momentum transfers *Q* covering the range 0.001–0.4 Å^–1^, where *Q* is defined as *Q* = (4π/λ) sin(θ/2) with θ being the scattering angle. All the samples were contained in fused silica cells with a path length of 1 mm. Data were corrected for the detector deadtime, flat field (using the scattering by 1 mm H_2_O), background (using boron carbide as an absorber), and transmission, using a beam monitor for normalization. The contribution from the empty cell was subtracted. The intensity level of water was used as a secondary standard to obtain intensities in the absolute scale. Data, being isotropic, were azimuthally averaged, leading to 1D profiles. To take into account the contribution of incoherent (*Q*-independent) scattering due to protons, a constant was subtracted from the spectra during the different fitting procedures. The data were analyzed by means of the open software packages SASview18 and SASfit employing the core–shell sphere model. This model provides the form factor, *P*(*Q*), for a spherical particle with a core–shell structure.

### Differential Scanning Fluorimetry

A Prometheus NT.84 nanoDSF device from NanoTemper Technologies GmbH (Munich, Germany) was used with standard glass capillaries (NanoTemper). Capillaries were filled with 10 μL of each sample. The excitation power was set to 30%, and the temperature ramp from 20 to 95 °C was run with a heat rate of 1 °C min^–1^. The intrinsic fluorescence was recorded at 330 and 350 nm. For the analysis, the signal obtained from the wavelength of the 330 nm channel was used. The protein–NP solutions were obtained by mixing the same volume of 10 mg mL^–1^ protein solution in D_2_O and 10 mg mL^–1^ NP dispersion to obtain a final concentration of 5 mg mL^–1^ of NPs and HSA each. The samples containing the mixture of NPs and HSA were incubated for 30 min at 37 °C and centrifuged at 20,000*g* again for 30 min. After centrifugation, the formed pellet was redispersed in the same amount of D_2_O. In the case of bare NPs and HSA, the final concentration was 5 mg mL^–1^ as well.

### Elastic Neutron Scattering

The ENS experiments were performed at the backscattering spectrometer IN13 at ILL, with an energy resolution of 8 μeV (integrating motions slower than approx. 80 ps) at ambient pressure at the temperatures of 280, 300, and 310 K. The sample solutions were loaded in flat sample holders of 1 mm thickness. The final concentration for native HSA and for HSA in all the mixtures was 5 mg mL^–1^. The NP–protein mixtures were centrifuged at 20,000*g* for 30 min and subsequently redispersed in D_2_O before the measurements to eliminate all free protein left in the solution. The elastic intensities, measured as a function of temperature, were reduced according to the standard procedure and normalized to vanadium.

## Results and Discussion

**Figure 1:**
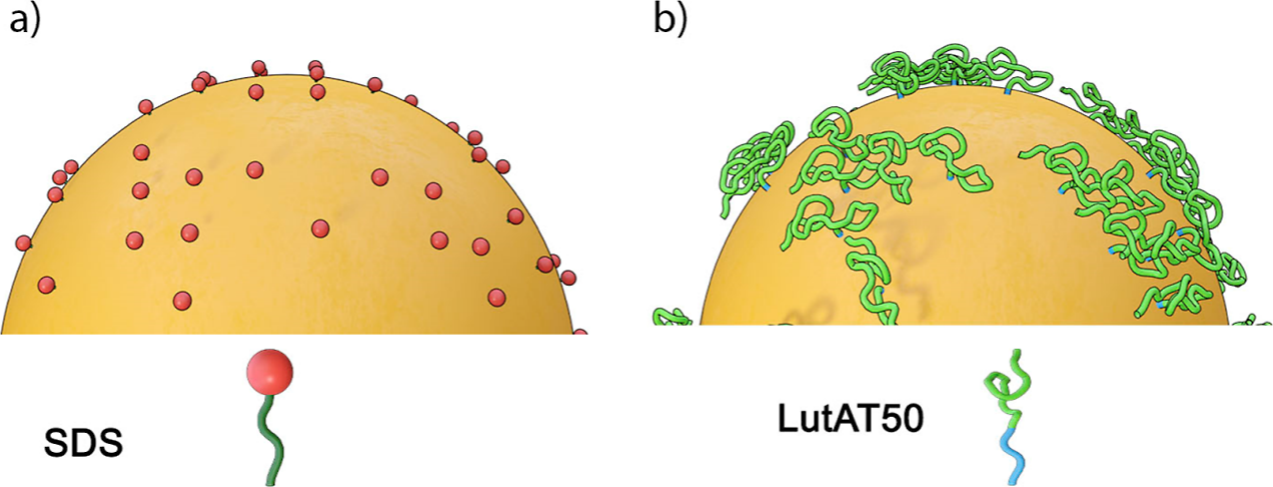
Schematic illustration of the surfactant distribution: (a) SDS and (b) LutAT50 on PS NPs surfaces.

To evaluate the PEG influence on protein adsorption, we compared NPs with a non-covalently PEGylated surface using the surfactant LutAT50 with NPs with a non-PEGylated surface (stabilized by SDS) (Figure 1).

Surfactants are key for NP synthesis and to ensure colloidal stability. SDS is an anionic surfactant composed of a hydrophobic tail and a negatively charged head group pointing toward the aqueous solution and providing electrostatic repulsion between the NPs. In low concentrations (i.e., below the quantification limit after extensive washing), SDS is well compatible with biological applications. On the other side, LutAT50 is a nonionic PEG-based surfactant constituted of a hydrophobic alkyl chain and a long hydrophilic PEG block that extends into the solution to a certain extent, providing steric stabilization. To study the influence of PEG on protein adsorption, this surfactant was selected as a straightforward way to functionalize NPs with PEG chains in the absence of another stabilizing agent (e.g., covalent PEGylation initially requires the addition of another charged surfactant during NP synthesis, which is then later mainly washed off, but minor amounts might remain). Thus, in this manner, the control over the type of functional moieties and, therefore, comparability between the two systems is better.

Two batches of PS NPs (stabilized either with SDS or with LutAT50) were prepared using styrene-*d*_8_ as a monomer with D_2_O as a dispersion medium. PS was selected as a NP model material because of its stability, reproducibility and control of process parameters. They are very well-studied and their physico-chemical properties (size, charge, etc.) can be precisely tuned, even with different surfactants for stabilization^20^ or changed solvents (D_2_O vs H_2_O). In addition, we know that the NP batches do not change (regarding colloidal stability or regarding surface composition) over long time frames (several months), which is necessary for performing studies with long experimental time frames, such as neutron scattering experiments. Both surfactants, SDS and LutAT50, are comparable in terms of stability, yielding similar size distributions of the NPs.

The size of the obtained NPs was measured by DLS (Table 1). The corresponding correlation functions and distributions are shown in Figures S1 and S2.

**Table 1:**
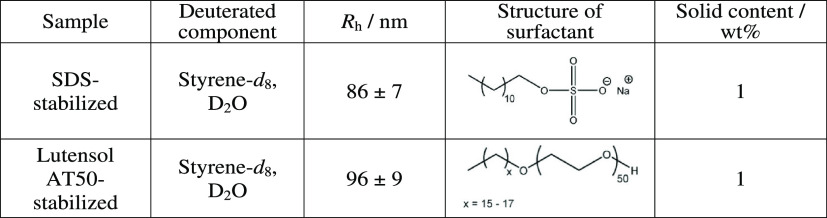
Characterization of the Investigated NP Batches

The obtained sizes of the NPs were similar within the experimental error range. It is important to remark that the NP size is highly influenced by the optimum surfactant concentration needed for the different stabilization mechanisms (ionic vs steric). Therefore, it is not trivial to obtain different batches of precisely the same size. To characterize the formed protein layer, both synthesized NP batches were incubated with HSA to allow protein adsorption. Then, the excess of free proteins was removed by performing centrifugation and washing steps (for details, see the Materials and Methods section). Further, these batches of NPs with adsorbed HSA and NPs without protein were used to perform SANS measurements.

### Small-Angle Neutron Scattering Experiments

**Figure 2:**
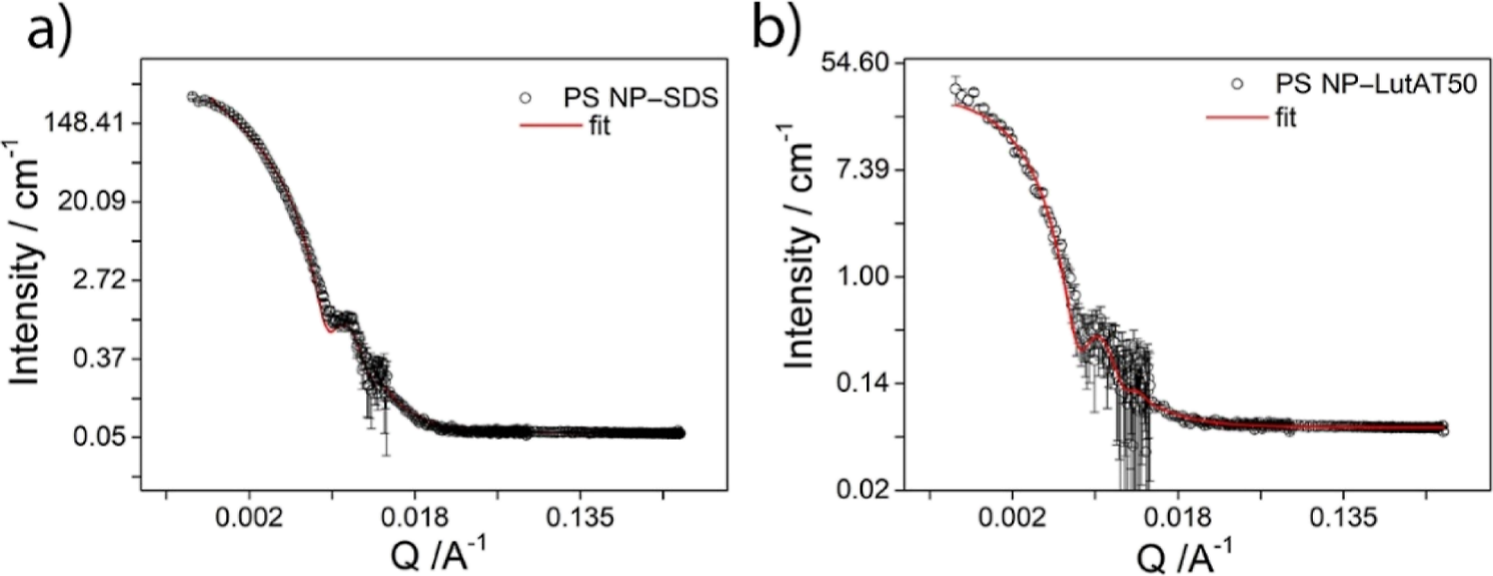
SANS experimental data with the corresponding best fitting curves superimposed to the PS NP–SDS (a) and PS NP–LutAT50 (b) experimental points.

First, stabilized NPs without proteins were analyzed. Figure 2 shows the scattering intensity versus the momentum transfer *Q* for PS NPs stabilized with SDS (a) and LutAT50 (b) without proteins.

To analyze the SANS profile, we used a core–shell sphere with a log-normally distributed radius of the core accounting for the PS latex and a constant shell thickness accounting for the surfactant coating. Interactions were neglected, that is, no structure factor was used. The scattering length density (SLD) (see the Supporting Information) of the core was imposed to the PS-*d*_8_ value, whereas the SLD of the shell was that of SDS or LutAT50. The scattering intensity of PS NP–LutAT50 was perfectly fitted using a core–shell model (Figure 2b). However, to analyze the PS NP–SDS sample, we used the fractal-core shell model that considers the scattering from fractal-like aggregates of building blocks of core–shell spheres.^21^ This model is based on the Teixeira model for the *S*(*Q*) of a fractal and a *P*(*Q*) for a core–shell model.^22^ It described the curve reasonably well in the whole *Q* range (Figure 2a), and the parameters are consistent with the simple core–shell model (where the low *Q* fit was not satisfying). Fitting with an SLD of the shell as the adjustable parameter was also performed but did not yield a reasonably good fit quality.

The radius (*R*_g_) obtained for PS NP–SDS was 650 Å (65 nm), whereas for PS NP–LutAT50, it was 693 Å (69 nm). As expected, the NP radii are slightly lower than those obtained using DLS (*R*_h_) (86 nm for PS NP–SDS and 96 nm for PS NP–LutAT50). The values obtained using DLS refer to the hydrodynamic radius and consider the hydration shell. Furthermore, the so-called ρ-ratio (ρ = *R*_g_/*R*_h_) is calculated for a homogeneous sphere to be 0.775.^23^ This value completely fits to SDS- and Lut-stabilized particles with ρ values around 0.7 in both cases. The inferred shell thickness of 2 Å for PS NP–SDS matches with the presence of SDS molecules, while the 3 Å shell thickness for PS NP–LutAT50 may suit LutAT50 with the hydrophilic PEG part in a mushroom conformation on the NP surface. The obtained values match the fact that the density of LutAT50 on the particle surface is rather low due to the washing and purification steps so that the average shell thickness might be lower than the local thickness of individual surfactant molecule patches. Moreover, the model applied (taking into account a small portion of aggregates) is in good agreement with the colloidal stability of the two particle types as the electrostatically stabilized NPs are slightly more prone to form aggregates than the sterically stabilized ones. The obtained radii and shell thicknesses from the analysis are summarized in Table 2.

**Table 2:** SANS Parameters of Radius and Shell Thickness of NPs before and after Incubation with HSA

NPs	radius (Å)	shell thickness (Å)
PS NP–SDS	650 ± 0.12	2
PS NP–LutAT50	693 ± 0.15	3
PS NP–SDS + HSA	650 ± 0.10	14
PS NP–LutAT50 + HSA	693 ± 0.10	18

**Figure 3:**
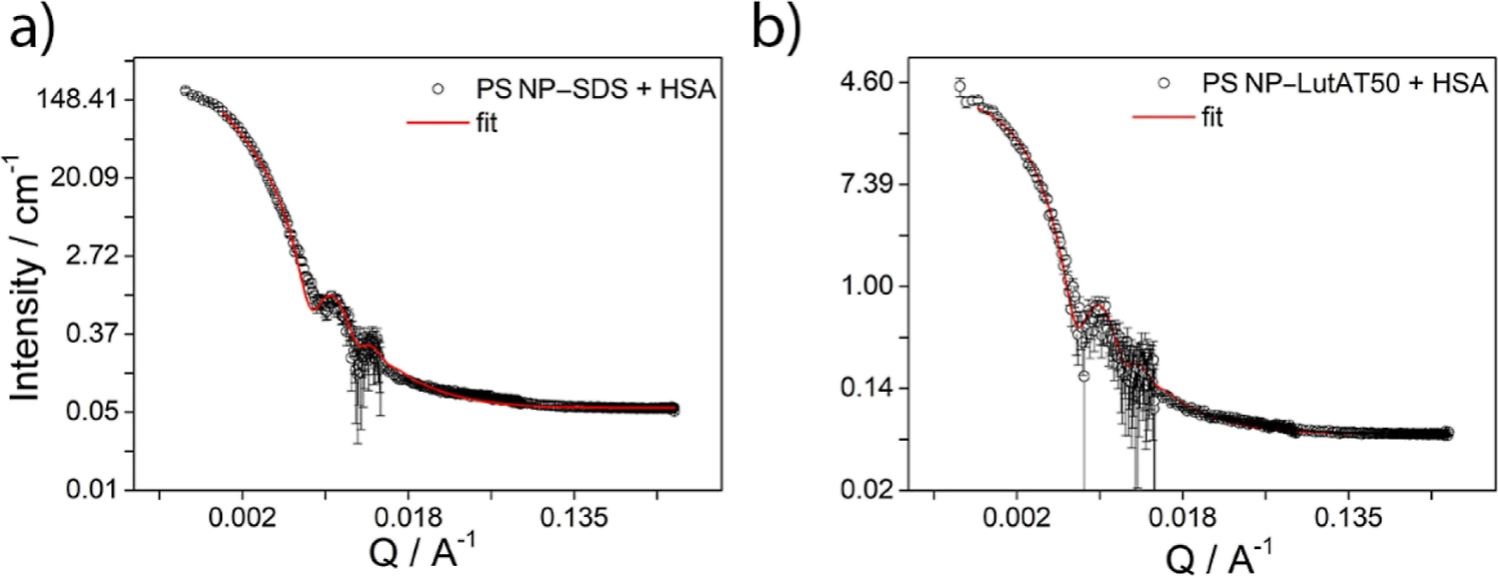
SANS experimental data with the corresponding best fitting curves superimposed to the experimental points for PS NP–SDS (a) and PS NP–LutAT50 (b).

After evaluating all pure components, including HSA and the surfactants (see Figures S3 and S4), the NPs were analyzed in the presence of adsorbed protein. To ensure that no significant amounts of free protein were in solution, the NP dispersions were washed by centrifugation and redispersion (see the Materials and Methods section). Figure 3 represents the scattering profile for PS NP–SDS (a) and PS NP–LutAT50 (b) after incubation with HSA, while the scattering profiles taken before washing are shown in Figure S5. The same models used for both pure NP batches were applied also after HSA incubation, that is, the fractal shell model for PS NP–SDS and a core–shell model for PS NP–LutAT50.

In both cases, the shell corresponding to the core–shell model was likely constituted by the surfactant, SDS or LutAT50 and HSA. However, it cannot be completely excluded that surfactant molecules were replaced by proteins during the adsorption process.

The SLD of the shell was imposed from the pure HSA since the surfactant SLD is 1 order of magnitude lower, whereas for the core, the value of the PS-*d*_8_ was selected. The inferred values are reported in Table 2. The same radii of pure NPs were retained for both NPs after the HSA incubation; that is, the radius was 650 Å (65 nm) for PS NP–SDS, whereas for PS NP–LutAT50, it was 693 Å (69 nm). Related to the shell thickness, the values obtained from the fit were 14 Å for PS NP–SDS and 18 Å for PS–NP–LutAT50. Thus, both shell thickness values are significantly lower than the thickness of native HSA in its smallest dimension (20 Å polar radius, see Figure S4 and Table S2), which hints at the fact that the HSA was not in its native state anymore. A total protein denaturation on PS NP–SDS may justify a shell thickness that is on average slightly thinner than for PS NP–LutAT50, where it could reveal partially native HSA.

The SANS experiments have highlighted the influence of the surfactants on the HSA adsorption. The results agree with a different protein conformation for both surfactants. The thickness for PS NP–SDS could fit with complete protein denaturation, whereas PS–NP–LutAT50 confers a lower impact on the adsorbed protein. It seems that the HSA configuration is retained slightly better after interaction with PS–NP–LutAT50, which might be due to the PEG chains on the NP surface. Therefore, the effect of the applied NP surface functionalization on protein adsorption requires a rigorous investigation, especially related to the native and denatured state of the proteins. To this aim, nanoDSF measurements were performed to determine the HSA conformation state after adsorption.

### Nano Differential Scanning Fluorimetry

**Figure 4:**
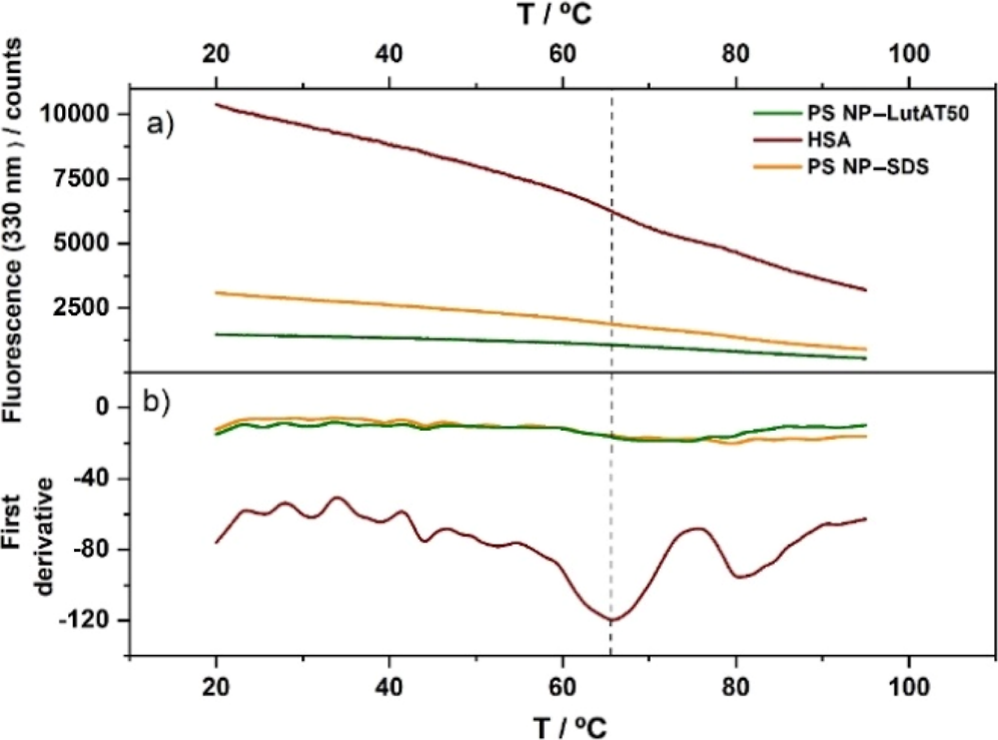
nanoDSF measurements of native HSA (red) and HSA after incubation with PS NP–SDS (orange) and PS NP–LutAT50 (green) in D_2_O showing the protein unfolding: (a) 330 nm fluorescence with the corresponding first derivative (b).

To further analyze the protein structure upon adsorption on the PEGylated and non-PEGylated surfaces, HSA was analyzed in detail by nanoDSF to observe the thermal unfolding of the protein. This method is based on the autofluorescence of tryptophan, which is a residue present in most proteins. Monitoring the heating of the protein, it is possible to detect the intrinsic fluorescence changes, which is influenced by its chemical environment. The changes are produced by the unfolding of the protein chain, thus allowing the determination of the melting temperature. The samples were heated (1 °C min^–1^) from 20 to 95 °C, and the intrinsic fluorescence intensity at 330 and 350 nm was recorded. Figure 4 shows the respective unfolding curve (a) and the associated first derivative at 330 nm (b). The native HSA (brown line) sample shows a considerable transition around 65.0 ± 1.3 °C, corresponding to the minimum in the first derivative. Additionally, a smaller second peak at 80 ± 1.5 °C is visible in the first derivative, which might be associated with a second domain. These structural transitions indicate unfolding or melting temperatures *T*_m_.

After incubation with HSA, the NP–protein complexes were also studied by nanoDSF. Here, as well, the free protein was removed by centrifugation. PS NP–SDS (orange curve) with HSA and PS NP–LutAT50 (green curve) with HSA display a significantly less pronounced transition than the native protein (see Figure S6 for more detailed information). However, it is worth mentioning that a very weak melting peak is always detected for HSA adsorbed on PS NP–LutAT50, but not on PS NP–SDS. These results are in line with the previous assumption: the HSA adsorbed was completely denatured on PS NP–SDS, whereas on the PS NP–LutAT50 surfaces, denaturation occurred as well but to a potentially lesser extent. This is in accordance with a larger shell thickness found in the SANS experiments.

### Protein Quantification

**Figure 5:**
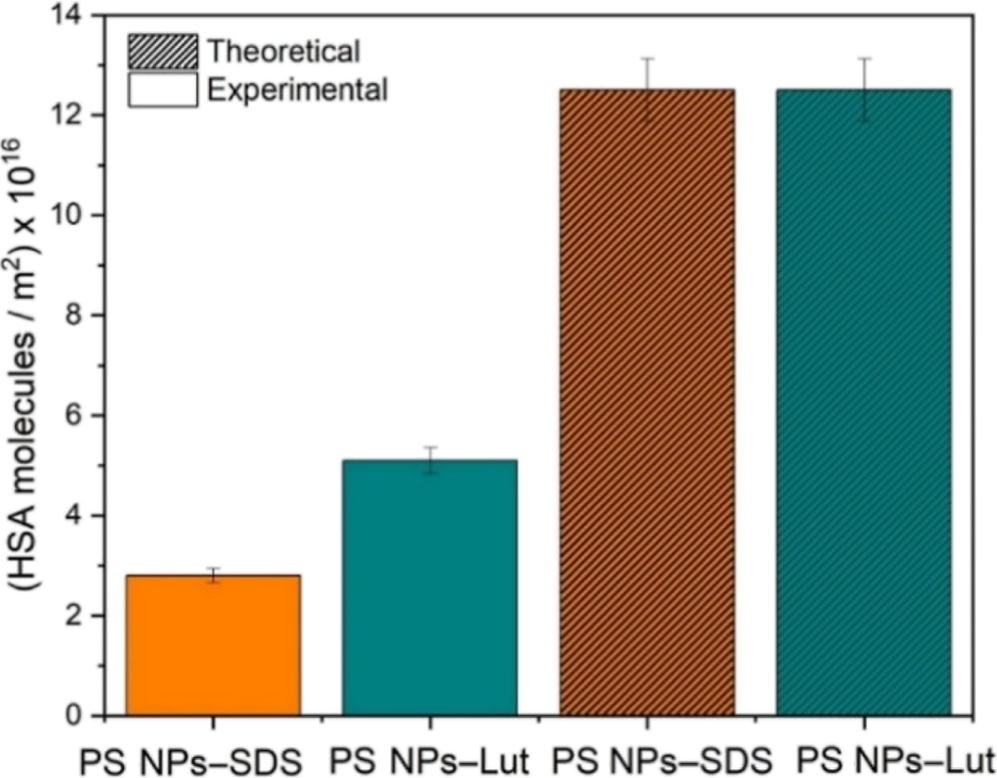
Adsorbed protein amount on NP surfaces experimental observed via a Pierce 660 nm assay and the theoretical value for maximum coverage calculated from HSA dimensions obtained in SANS experiments.

The total protein mass adsorbed on both NP surfaces was detected via a Pierce 660 nm quantification assay to further characterize the protein–surface interaction. Samples were prepared as explained in the Materials and Methods section and analyzed photometrically. The amount of protein obtained was normalized to the NP surface area and is shown in Figure 5.

It was estimated that the experimentally obtained surface coverage by HSA was roughly double for PS NP–LutAT50 compared to that for PS NP–SDS with values of 5.1 × 10^16^ and 2.8 × 10^16^ HSA molecules/m^2^, respectively. Then, the experimental values were compared to the theoretical maximum coverage based on the dimensions of the native HSA determined by SANS (40 × 20 Å), assuming a completely dense packing. The amount of HSA on the NP surface was 4.4 times lower than the theoretical value for PS NP–SDS, whereas for PS NP–LutAT50, it was only 2.5 times lower.

The fact that the experimental values for the protein amount are well below the theoretical maximum coverage agrees with the finding that HSA molecules on the NP surfaces are partially or fully denatured. In that case, they need a larger area to spread out on the surface and, therefore, lead to fewer molecules per square meter. Again, we can confirm the trend that HSA on the PEGylated surface seems to be less denatured as more HSA molecules can be packed in the adsorbed layer. Furthermore, opposite to the general thoughts about PEG decreasing unspecific protein adsorption, the amount of HSA adsorbed was double for PS NP–LutAT50 than for PS NP–SDS. In this context, it is essential to highlight that the protein adsorption and the correlated stealth effect are determined by the interaction with other proteins. However, in this case, the study of the single protein HSA implies that there was no competition with other proteins for the surface interaction.

### Elastic Neutron Scattering

Finally, ENS experiments were carried out to confirm our findings regarding the protein conformation. From these experiments, it is possible to obtain information about the protein dynamics on the NP surface that can be related to the protein conformational state.

**Figure 6:**
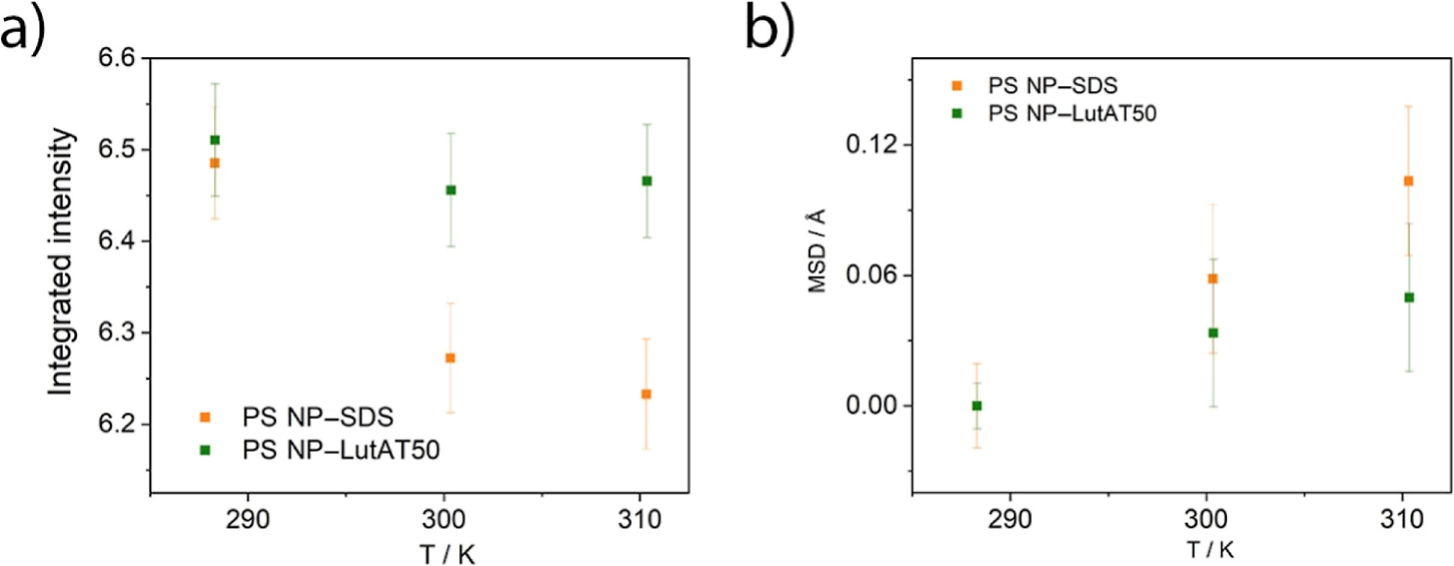
ENS results. (a) Integrated intensity vs temperature and (b) MSD vs temperature for PS NP–SDS (orange squares) and PS NP–LutAT50 (green squares).

Figure 6a shows the integrated elastically scattered intensity versus temperature plot where the orange line corresponds to the PS NP–SDS and the green one corresponds to PS NP–LutAT50 after incubation with HSA. The decrease in temperature of the elastic intensity, generally observed,^24,25^ is governed by the thermal activation of movements in the time domain above 80 ps. The temperature dependence is more pronounced in the presence of SDS. The lower the elastic intensity, the more pronounced the active dynamics, pictured in terms of flexibility. The results suggest that the HSA molecules on the NPs stabilized with SDS are more flexible than those stabilized with LutAT50.

Figure 6b shows the mean square displacement (MSD) inferred from the data. The MSD temperature dependence (slope) can be associated to the flexibility of the system, while the amplitude of the fluctuations is related to the explored space by the atoms. Since the MSD inferred for the HSA protein on PS NP–LutAT50 is lower than on PS NP–SDS, it is tempting to affirm that the adsorbed HSA is more confined in the presence of LutAT50 rather than SDS. More pronounced flexibility and lower confinement of the HSA in the presence of SDS support the previous speculation of a partially or fully denatured protein. A denatured protein presents higher mobility than its native counterpart because of the loss of the secondary structure.^26,27^ Our results suggest that the surface functionalization of PS NPs with PEG-based surfactants influences the nature of the adsorbed protein layer. Although individual experiments cannot state this conclusively, these results are consistent and provide enough evidence to support our hypothesis. The latter suggests that the HSA structure changes after its adsorption on the NP surface with a lower impact for PS NP–LutAT50, probably due to the presence of PEG groups. The secondary structure of HSA was shown to control the cell surface receptors used when it is adsorbed on NPs.^28^ Therefore, it is crucial to investigate the role of the native structure of proteins on biomedical application. In general, surface functionalization with PEG—or proposedly similarly hydrophilic biocompatible polymers—seems beneficial for preventing the induction of the immune system response due to protein denaturation.

## Conclusions

This work demonstrates the influence of PEG chains on protein adsorption on NP surfaces. PEG chains were introduced using the PEG-based surfactant LutAT50. Its effect was compared with that of NPs stabilized with the anionic surfactant SDS.

The use of neutron scattering techniques allowed obtaining structural information about the formed protein layer. It appears to be less flexible on the PEGylated surface. Likewise, through nanoDSF measurements, it was confirmed that HSA completely lost the native structure after adsorption on SDS-stabilized surfaces. On the LutAT50-stabilized surface, this effect was slightly less pronounced because of the PEG chains on the NP surface.

The combination of the results reported here indicates that the loss of protein conformation is lower on PEGylation surfaces. This effect is expected to be even stronger at higher PEG densities due to a thicker hydrophilic barrier to hinder the protein interaction with the surface.

Understanding the relationship between the protein structure and function is critical to the design and success of biomedical applications. However, it remains one of the central aims still under discussion. Therefore, this work contributes to improving the knowledge about the way PEGylated surfaces influence the protein adsorption to develop new systems based on the native structure of the proteins.

## Supporting Information Available

DLS data for NP dispersions, SANS parameter definition and values, SANS experimental data of samples without centrifugation, and experimental data of nanoDSF (PDF)

## Abbreviations

DSF: differential scanning fluorimetry
ENS: elastic neutron scattering
HSA: human serum albumin
LutAT50: Lutensol AT50
NP: nanoparticle
PEG: poly(ethylene glycol)
SANS: small-angle neutron scattering
SDS: sodium dodecyl sulfate
