# Supplementary material for: Poly(ethylene glycol)-Based Surfactant Reduces the Conformational Change of Adsorbed Proteins on Nanoparticles
Source: Biomacromolecules. 2022 Sep 9;23(10):4282–8. doi: 10.1021/acs.biomac.2c00744 (PMC9554902; doi:10.1021/acs.biomac.2c00744)
Supplement: Supplementary file 1 — bm2c00744_si_001.pdf [file bm2c00744_si_001.pdf]

Supporting Information

Poly(ethylene glycol) based surfactant reduces conformational change of adsorbed proteins on nanoparticles

María Martínez-Negro,[a] Daniela Russo,[b] Sylvain Prévost,[c] José Teixeira,[d] Svenja Morsbach,[a] and Katharina Landfester [a]

[a] Max Planck Institute for Polymer Research, Ackermannweg 10, 55128 Mainz

[b] Consiglio Nazionale delle Ricerche (Instituto Officina dei Materiali) and INSIDE@ILL c/o Institut Laue-Langevin, 38042 Grenoble, France

[c] Institut Laue-Langevin, 38042 Grenoble, France

[d] Université Paris-Saclay, Laboratoire Léon-Brillouin, UMR12 CEA-CNRS, CEA-Saclay, F-91191 Gif-sur-Yvette CEDEX, France

Table of contents

1. DLS measurements of NPs
2. Small angle scattering parameters
    - Parameter definition
    - Parameter (SLD and radius) values
    - SANS profile of pure HSA
3. SANS profiles without centrifugation
    - PS-NP-SDS and PS-NP-LutAT50 profile
4. Elastic neutron scattering (ENS)
5. Differential scanning fluorimetry (DSF)

1. DLS measurements of NPs

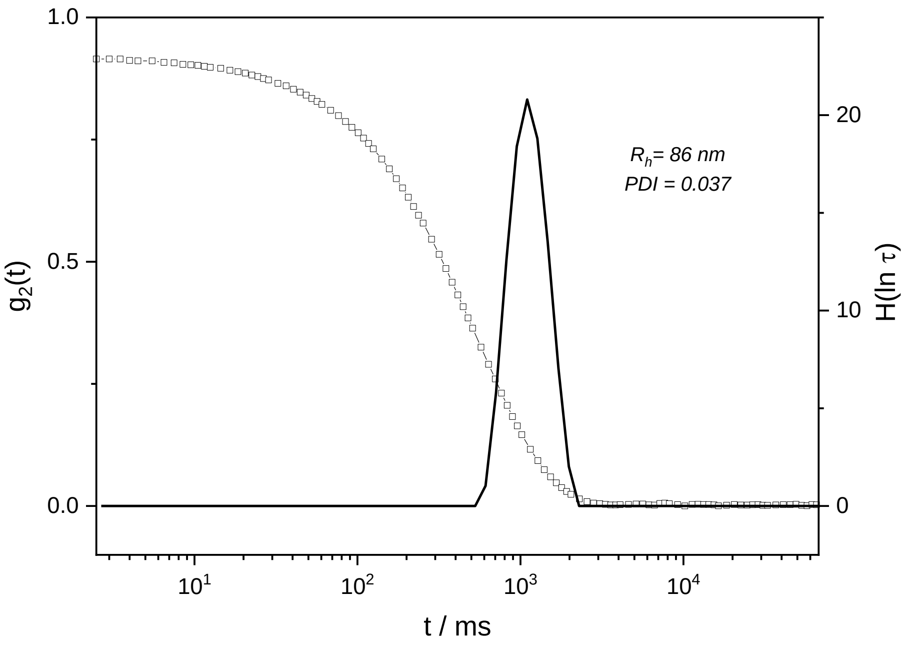

Figure S1. Correlation function g_2(t) of PS-NP-SDS for a scattering angle of 90° together with the corresponding distribution of relaxation times H (ln τ).

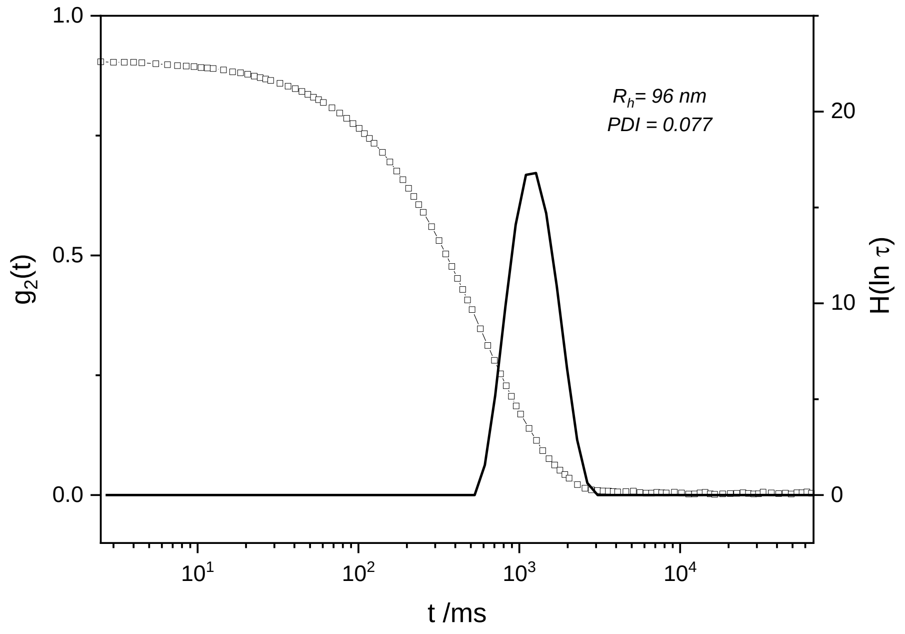

Figure S2. Correlation function g_2(t) of PS-NP-LutAT50 for a scattering angle of 90° together with the corresponding distribution of relaxation times H (ln τ).

2. Small angle scattering parameters

The measured intensity I(Q) as a function of the wave vector Q, can be expressed as:

$$I(Q) = nP(Q)S(Q) + B \tag{S1}$$

where *n* is the number density of the particles, *P(Q)* is the form factor characteristic of the specific size and shape of the scatterers and *S(Q)* is the structure factor that accounts for the inter-particle interaction. *B* is a constant that represents the incoherent background scattering, mainly from the hydrogen atoms.

The NP scattering data were fitted using the spherical core shell model. This model provides the form factor, *P(Q)*, for a spherical particle with a core-shell structure[1]. The core and the solvent scattering density where imposed during the fitting procedure.

Related to the analysis, one of the main parameters of SANS is the scattering length density (SLD) which is a measure of the scattering power of a material. It is calculated according to equation (S2) where b_i are the nuclei scattering lengths of each of the n atoms in the molecule and υ_m is the molecular volume:

$$SLD = \frac{\sum_{i=n}^{n} b_i}{\upsilon_m} \tag{S2}$$

Molecular volume was calculated as following:

$$v_m = \rho_{molecule} \frac{N_A}{M_w} \tag{S3}$$

where N_A corresponds to Avogadro's Number (6.023x 10^{23} mol^{-1}).

The different SLD values calculated and used to analyze SANS measurements are collected in Table S1.

Table S1. SLD values calculated of each molecule and imposed in the analysis.

	Polystyrene-d8	SDS	LutAT50	HSA	D_2O
SLD / 10^{-6} Å^{-2}	6.1	0.33	0.48	3.9	6.3

SANS measurements were also carried out on pure surfactants as a control. SDS and LutAT50 solutions were analyzed above their respective critical micelle concentrations (CMC), 8.1 mM and 2.10^{-9} mM respectively. At CMC value, surfactants form micelles and this would make possible to obtain the characteristic profile arising from the presence of micelles in solution. The scattering profiles are shown in Figure S3.

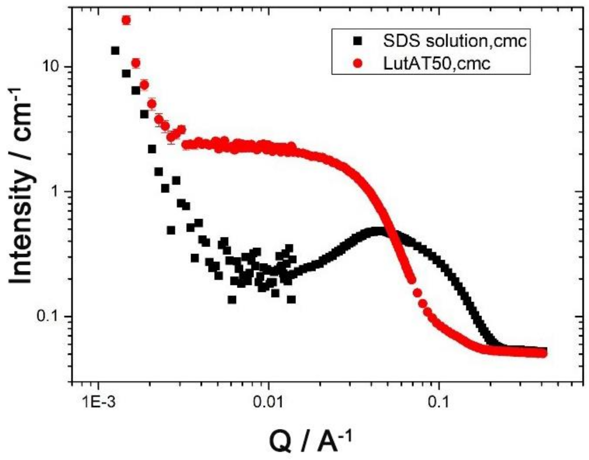

Figure S3. SANS experimental data for the SDS (black profile) and the LutAT50 (red profile) at CMC.

When we compare the scattering profile of the SDS and Lutensol solution, at high concentration, we observe a different behavior. SDS has a characteristic profile arising from the presence of micelles in solution. LutAT50 could be analysed in terms of a star-polymer or polymer micelle model. Moreover, the hydrodynamic radius of the surfactant micelles was determined by dynamic light scattering (DLS). For SDS, a size of the hydrodynamic radius R_h = 3.8 nm was obtained, while for LutAT50 micelles, the radius was found to be R_h = 6.5 nm. This is in line with the expectations as LutAT50 has a higher molecular weight (M_w = 2460 g mol^{-1}) than SDS (M_w = 288.4 g mol^{-1}) and especially a much larger hydrophilic part.

As a third pure component, the native protein, human serum albumin (HSA), was analyzed and the scattering profile was adjusted to an ellipsoidal model in agreement with values previously estimated by neutron scattering and in agreement with those reported in the literature (Figure S4). The dimensions obtained for HSA (Table S2) were 20 Å x 40 Å for polar and equatorial radius respectively.

Table S2. Polar and equatorial radius of HSA.

**Protein**	**RADIUS POLAR / Å**	**RADIUS EQUATORIAL / Å**
HSA	20	40

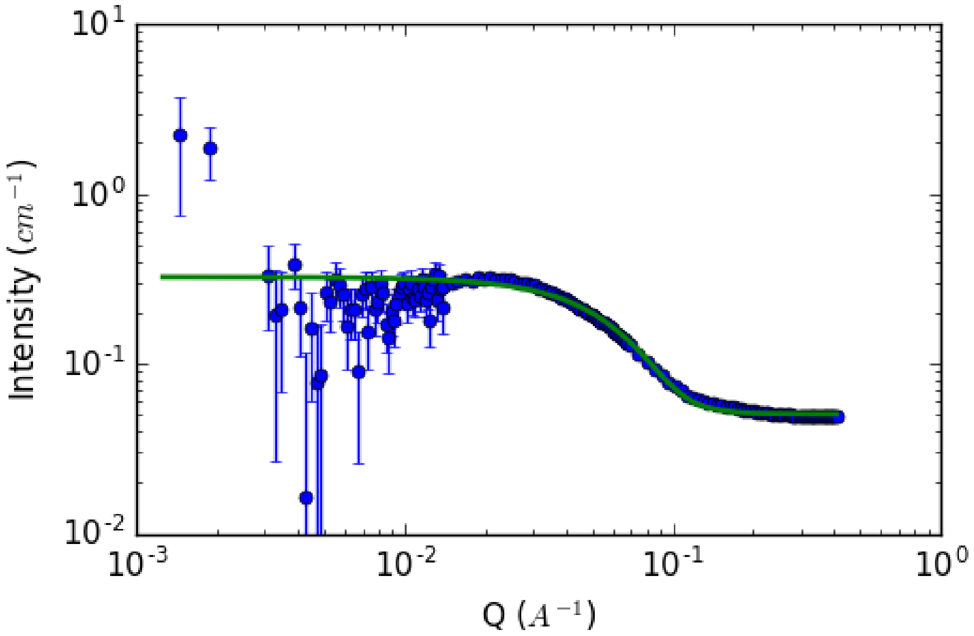

Figure S4. SANS experimental data for HSA with the corresponding best fitting curves superimposed to the experimental points.

3. SANS profiles without centrifugation

Additionally, both NPs, PS-NP-SDS and PS-NP-LutAT50, were also measured without centrifugation after incubation with HSA. Figure S5 shows the SANS profiles for NPs stabilized with SDS (a) and LutAT50 (b). At high Q it is possible to observe a bump corresponding to the protein signal. Since the samples were not centrifuged, the excess of HSA in solution covers the effect of the shell.

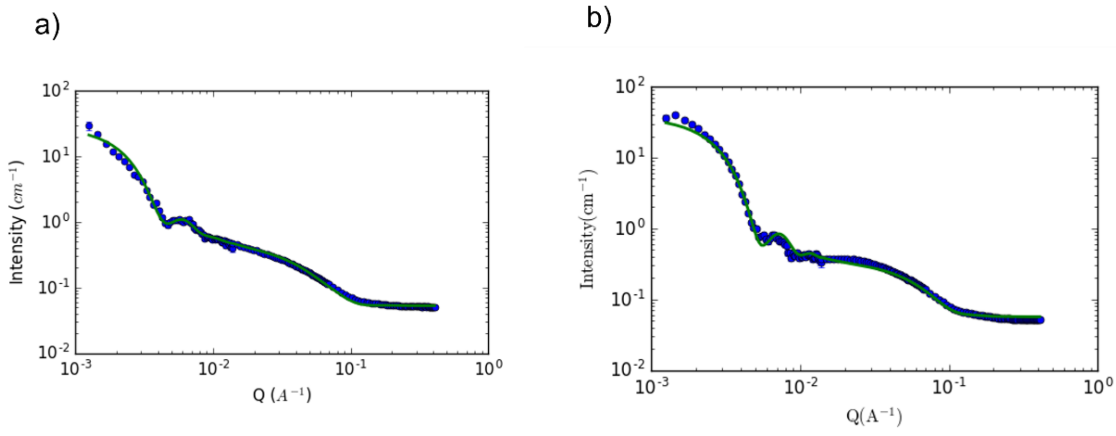

Figure S5. SANS experimental data with the corresponding best fitting curves superimposed to the experimental points for PS-NPS-SDS (a) and PS-NP-Lut (b) without centrifugation.

4. Elastic neutron scattering

Elastic scans were carried out using the so-called fixed window scans, in which only the elastic intensity, convoluted with the instrumental function, is measured as a function of the temperature. Typically, an important decrease of the intensity marks the onset of a relaxation process on the time scale of the instrument. The evaluation of the Q dependence of the scattering intensity gives access to the mean square displacement of the hydrogen atoms as a function of temperature. The intensity can be written as:

$$I\,(Q) = I\,(0)e^{(-<u^2>Q^2/3)} \qquad \text{(S4)}$$

$<u^2>$ might be interpreted as the average radius of the region dynamically occupied by the hydrogen atoms. At low temperature $<u^2>Q^2$ corresponds to the vibrational Debye Waller factor, while rotational and translational motions will contribute when increasing the temperature

5. Differential scanning fluorimetry

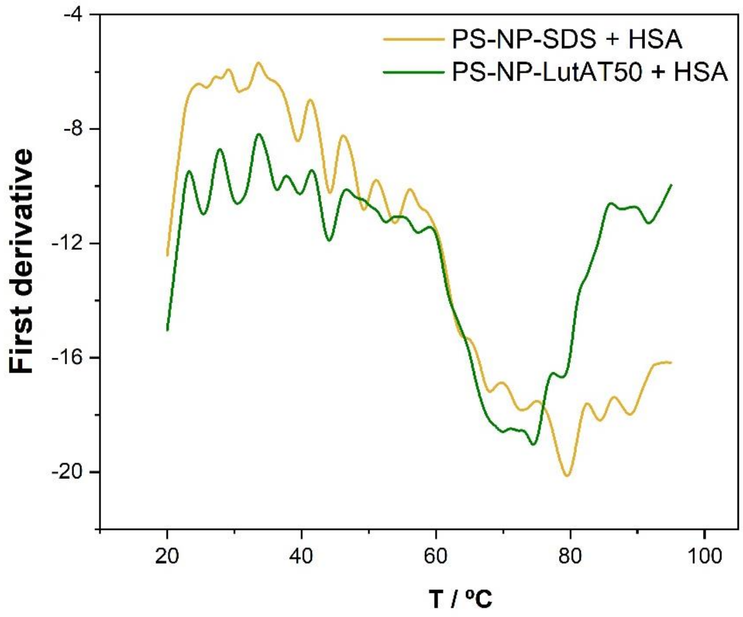

Figure S6. Differential scanning fluorimetry measurements (nanoDSF) of native HSA after incubation with PS-NP-SDS (orange) and PS-NP-LutAT50 (green) in D_2O showing the protein unfolding.

6. References

1. A. Guinier, G. Fournet and K. L. Yudowitch, 1955.
